# Cartilage Framework Fixation with 2-Octyl Cyanoacrylate in a Prelaminated Radial Forearm Free Flap Total Auricular Reconstruction

**DOI:** 10.7759/cureus.6389

**Published:** 2019-12-15

**Authors:** Cara Reitz, Chris England, Owen N Johnson

**Affiliations:** 1 Surgery, William Beaumont Army Medical Center, El Paso, USA; 2 Otolaryngology, Proliance Surgeons, Mount Vernon, USA; 3 Surgery, Uniformed Services University of the Health Sciences, F. Edward Hébert School of Medicine, Bethesda, USA

**Keywords:** ear reconstruction, cartilage framework, prelaminated, microsurgery, head and neck, reconstruction, adhesives, dermabond, cyanoacrylate, fixation

## Abstract

Autologous total auricular reconstruction requires an intricately sculpted, curved, and stacked cartilaginous framework implanted under healthy vascularized tissue. The ideal fixation technique would be readily available, easy to deploy, and free of complications. Commonly used sutures can fracture fragile pieces, inadequately maintain shape, or migrate. Steel wire can erode through soft tissues, extrude, fracture, or become infected. We successfully used 2-octyl cyanoacrylate alone to fixate an autologous costal cartilage framework designed for a total auricular reconstruction in an adult trauma patient. We had no sutures or wires in the final construct. The key aspects of our technique included the following: use of small aliquots, application only at cartilage-to-cartilage interfaces, use of temporary shaping (needles and lasso sutures), and avoidance of excess spillage of adhesive on any portion that would directly contact soft tissue. The framework was implanted into a prelaminated radial forearm free flap, which was then transferred to the head and neck region at a second stage. At two-year follow-up, the framework held satisfactory shape without any complications such as resorption, exposure, or infection.

## Introduction

A detailed cartilaginous framework must be intricately sculpted, curved, stacked, and then fixated in autologous total auricular reconstruction. Sutures can fracture fragile pieces of cartilage, leading to ingrowth and resorption. Steel wire can extrude. Rhinoplasty surgeons have cautioned against using bioadhesives to stabilize cartilage grafts due to prolonged inflammation and infection, although data have actually been mixed or confounded by other variables [1]. We report successfully employing 2-octyl cyanoacrylate (Dermabond, Ethicon, Somerville, NJ) as the sole fixation method of a costal cartilage auricular framework in a prelaminated radial forearm free flap (RFFF) without complication.

## Case presentation

A 20-year-old female suffered traumatic avulsion of the left auricle with diffuse hypertrophic scarring of the surrounding soft tissue areas. External auditory canal cicatricial stenosis was associated with subjective early hearing loss. Treatment of the stenosis was indicated. The patient considered prosthetics, but free tissue transfer was deemed the best option. Local soft tissues were inadequate as reliable flap donors or for stable coverage of osseointegrated hardware.

We discussed a multistage total reconstruction using an autologous costal cartilaginous framework prelaminated into a right RFFF. This multimodality plan also included targeted triamcinolone acetonide injections and meatoplasty.

**Figure 1:**
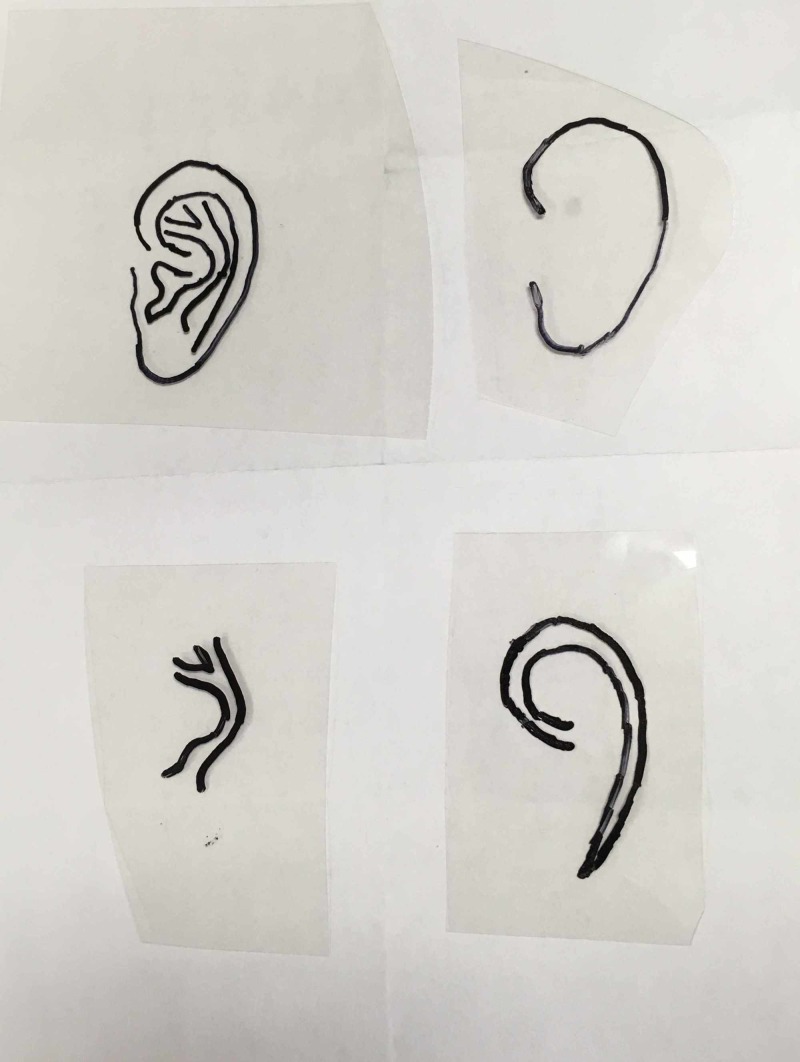
Mirrored templates based on the unaffected ear used in framework design.

Cartilage was harvested from the right synchondrosis. The framework design was based on the unaffected contralateral ear (Figure 1).

The framework was constructed using a modified Nagata technique. To ensure adequate projection, multiple pieces were stacked. Driving needles and sutures into the fragile pieces would have risked fracture of the precious cartilage.

**Figure 2:**
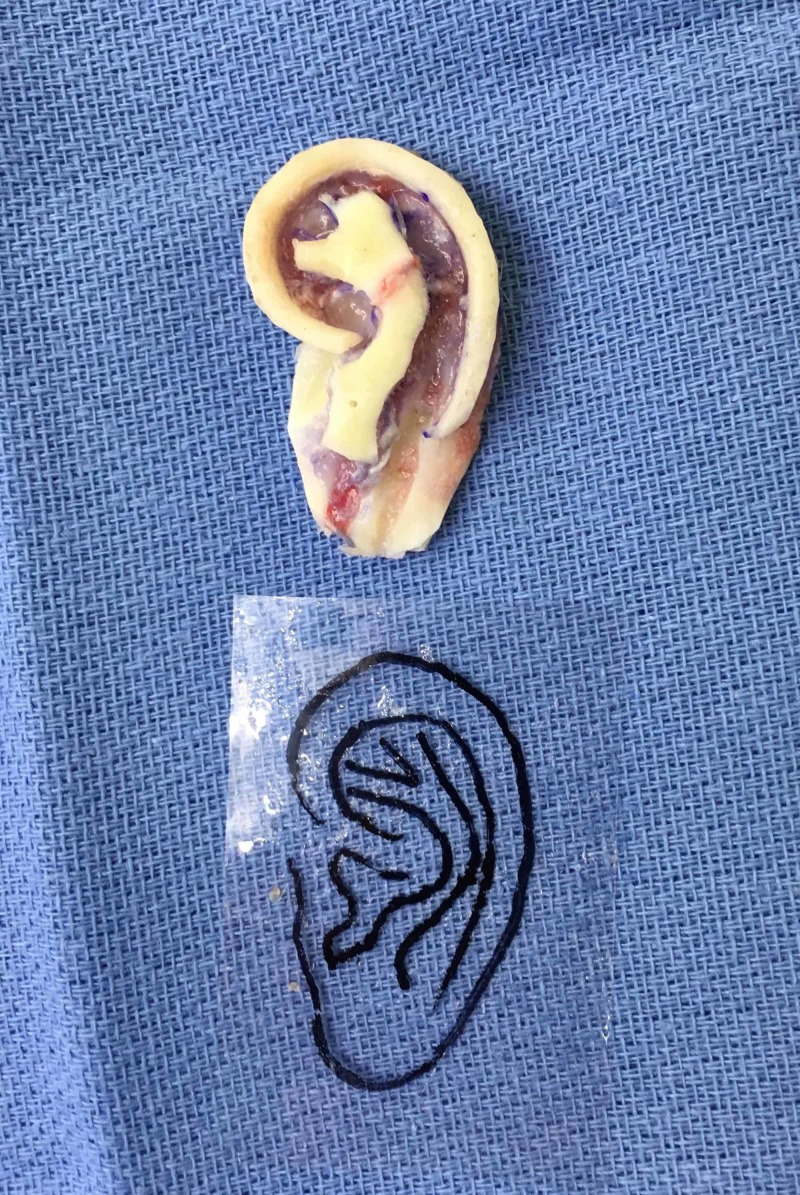
Intraoperative image of autologous costal cartilage framework prior to further carving and refinement ahead of implantation into right forearm. No sutures or wires were used for fixation.

We decided to use fine syringe needles for initial shaping along the perimeter of the framework, similar to concrete stakes. 5-0 monofilament polypropylene sutures were used for lasso pieces as needed. We applied small aliquots of Dermabond to cartilage-to-cartilage interfaces. We took care to avoid spilling excess material onto parts of the framework that would contact soft tissue (Figure 2).

**Figure 3:**
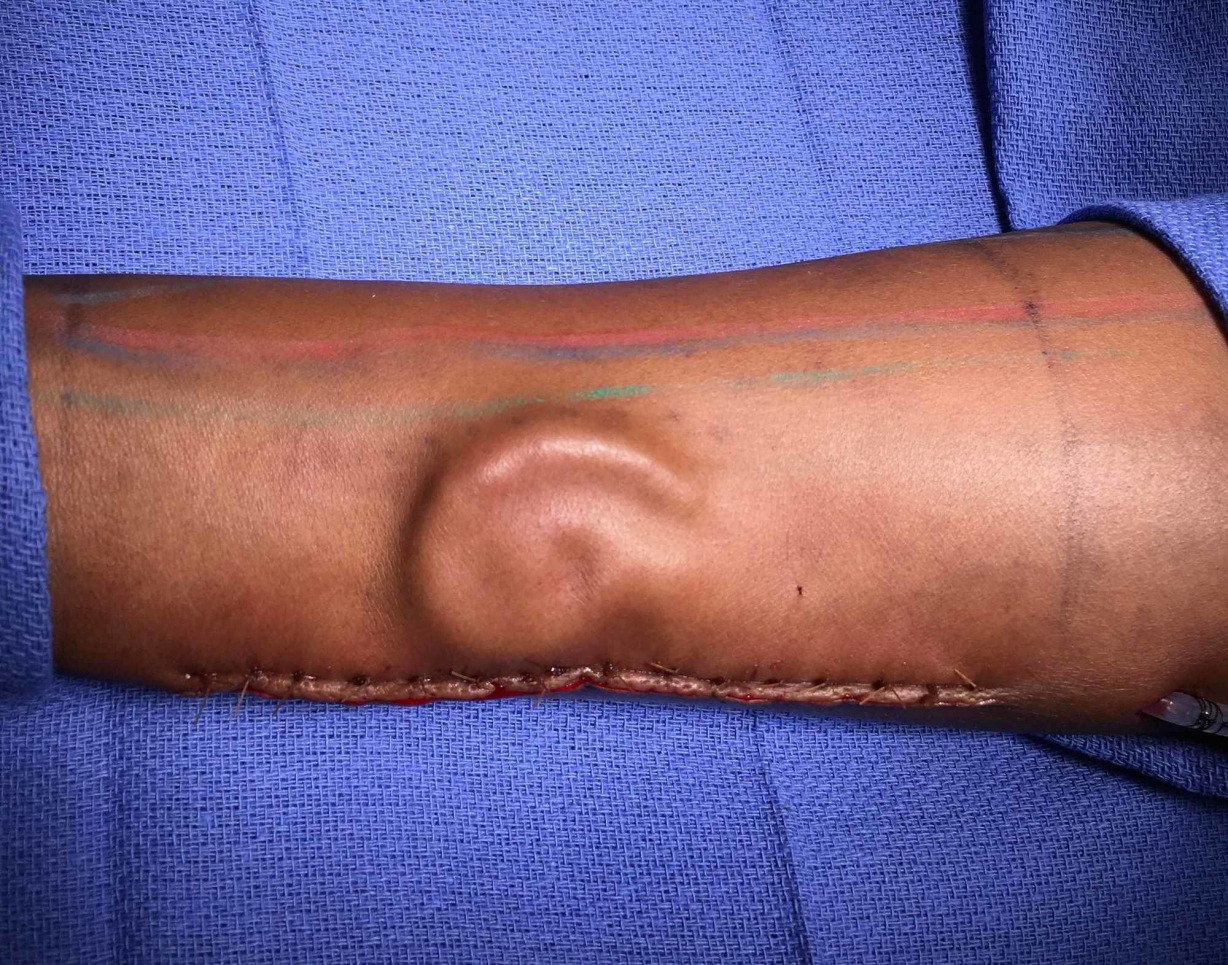
The framework was implanted into the suprafascial plane of a right radial forearm free flap and allowed to mature for one year.

Once completed, the framework was intact and solid, holding good shape without the need for any sutures or wires. This was implanted into the suprafascial plane of a planned RFFF, ulnar to the flexor carpi radialis tendon, through an ulnar skin incision. Sensory nerves were preserved (Figure 3).

There were no complications, and the framework matured in the forearm for one year. It maintained its shape with no resorption. There was normal edema and inflammation, but over time the soft tissue contracted over the framework and definition improved. The patient had no seroma, hematoma, tissue breakdown, infection, or foreign body reaction.

**Figure 4:**
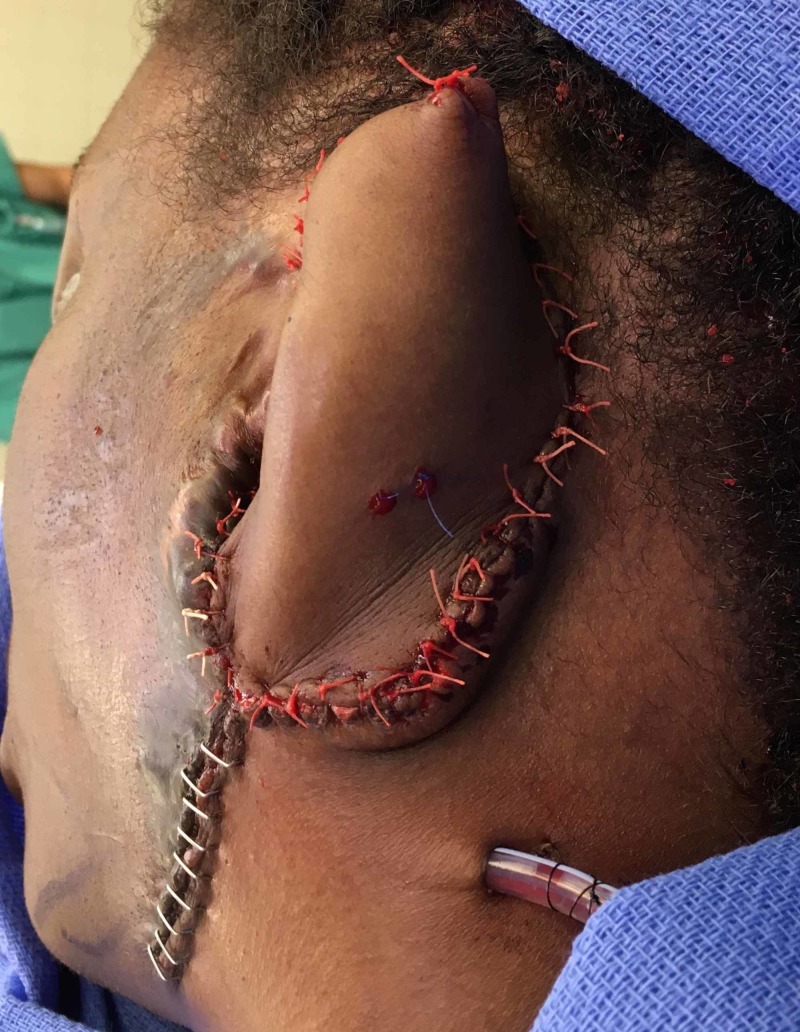
On-table appearance of the prelaminated flap upon transfer to the head and neck region.

At the second stage, the right RFFF was transferred to the left facial vessels. Redundant skin and fascia were used at inset to elevate the construct from the mastoid. The reconstructed ear’s height, lateral projection, and other measurements such as neo-helix-mastoid angle, neocephaloauricular and neo-scaphaconchal angles were carefully matched to the uninjured side (Figure 4). At two-year follow-up, the flap, with its intact framework, was healthy and ready for additional debulking and refinement.

## Discussion

Total ear reconstruction is considered for traumatic avulsion, burns, microtia, or following tumor extirpation [2-5]. Planning must consider framework, soft tissue coverage, and patient characteristics and goals.

Prosthetics that rely on osseointegrated hardware require healthy bone and stable soft tissue coverage. Porous polyethylene is available as a synthetic framework, but complications include failed osseointegration, delayed healing, dehiscence, device fracture, infection, and need for repeat operations [6,7].

Long et al. reviewed complications after autologous cartilage auricular reconstruction [8]. These included unsatisfactory results, wire or suture extrusion, and cartilage resorption as well as infection, hematoma, skin necrosis, framework exposure, framework fracture, and facial nerve injury. Their review noted that wire fixation was correlated with higher rates of extrusion, especially at longer follow-up intervals.

Extrusion and infection are also possible with suture techniques, especially when permanent sutures are used. Sakamoto compared different fixation materials and found the lowest complication rate to be with absorbable sutures [9].

Ma et al. described using 5-0 steel wires to suture and splice their sculptured frameworks for microtia reconstruction [4]. They noted the need for caution to avoid fracturing the cartilage, which can occur when passing suture or wire, and often is worse when passing through multiple layers. When fractured, connective tissue is allowed to infiltrate the cartilage and can ultimately lead to resorption, affecting functional and aesthetic outcomes [4,10]. The Nagata method utilizes approximately 80 pieces of 38-gauge stainless steel wire [11].

We were interested in alternative fixation methods that could avoid the need for sutures or wires. Dermabond has been used in skin closures, ossicular reconstruction, laryngeal repairs, reconstructive rhinoplasty, dental reconstruction, radiologic embolization, dural repairs, endoscopic treatment of enterocutaneous fistulas, management of pulmonary air leaks, and corneal perforation repairs [10,12,13]. It is a long-chain adhesive that exhibits less tissue toxicity, confers more microbial resistance, and contains additional stabilizers for improved tensile strength compared to earlier-generation versions [13,14].

Gall et al. reported postoperative septal abscess following use of Dermabond in septorhinoplasty [1]. They, however, used a polydioxanone plate secured with both Dermabond and suture in their repair, which may confound any attribution of infection to the use of adhesive alone. Min and Jang also found a higher complication rate when using adhesives for tip grafting in rhinoplasty [15]. Open rhinoplasty is known to be associated with significant swelling, especially when foreign materials are used under a thin skin and soft tissue envelope. The authors commented that their complications were often observed when multiple layers of cartilage were required, larger amounts of adhesive were used, and high amounts were spilled into surrounding tissues.

Early animal studies showed N-octyl cyanoacrylate elicited similar inflammatory responses and had similar migration propensity as sutures [10,12]. Its advantages include ease of application, decreased damage to cartilage, and removal of needles from the operative field. In areas where suturing is not accessible such as in posterior cricoid fractures, Dermabond fixation of cartilage shows a definite advantage [16]. Seo et al. had favorable results with no significant protrusions, dislocations, or unusual inflammation after rhinoplasty in Asian patients [14]. Dabb et al. also reported good outcomes with no complications in a cohort of nine patients [13].

## Conclusions

The use of 2-octyl cyanoacrylate was successfully used in the construction of our auricular framework for prelaminated free flap total ear reconstruction, chosen for its ease of application, microbial resistance, and tensile strength. We avoided using any sutures or wire, and did not fracture any cartilage pieces. Postoperative healing was not negatively impacted compared to standard fixation, and the patient experienced no infectious or excessive inflammatory complications. The cornerstones of our technique included the following: use of small aliquots, application only at cartilage-to-cartilage interfaces, use of temporary shaping (needles and lasso sutures), and avoidance of excess spillage of adhesive on any portion that would directly contact soft tissue.
